# Hyperglycemia induced by pasireotide in patients with Cushing’s disease or acromegaly

**DOI:** 10.1007/s11102-016-0734-1

**Published:** 2016-07-12

**Authors:** Julie M. Silverstein

**Affiliations:** Division of Endocrinology, Metabolism and Lipid Research, Washington University School of Medicine, St Louis, MO USA

**Keywords:** Acromegaly, Cushing’s disease, Hyperglycemia, Pasireotide

## Abstract

**Purpose:**

Cushing’s disease (CD) and acromegaly are characterized by excessive hormone secretion resulting in comorbidities such as impaired glucose metabolism, diabetes and hypertension. Pasireotide is a new-generation, multireceptor-targeted somatostatin receptor ligand approved for CD (subcutaneous [SC] injection formulation) and acromegaly (long-acting release [LAR] formulation). In clinical studies of pasireotide, hyperglycemia-related adverse events (AEs) were frequently observed. This review highlights differences in reported rates of hyperglycemia in pasireotide trials and discusses risk factors for and management of pasireotide-associated hyperglycemia.

**Methods:**

Clinical trials evaluating pasireotide in patients with CD or acromegaly were reviewed.

**Results:**

The frequency of hyperglycemia-related AEs was lower in patients with acromegaly treated with pasireotide LAR (57.3–67.0 %) than in patients with CD treated with pasireotide SC (68.4–73.0 %). Fewer patients with acromegaly treated with pasireotide LAR discontinued therapy because of hyperglycemia-related AEs (Colao et al. in J Clin Endocrinol Metab 99(3):791–799, 2014, 3.4 %; Gadelha et al. in Lancet Diabetes Endocrinol 2(11):875–884, 2014, 4.0 %) than did patients with CD treated with pasireotide SC (Boscaro et al. in Pituitary 17(4):320–326, 2014, 5.3 %; Colao et al. in N Engl J Med 366(10):914–924, 2012, 6.0 %). Hyperglycemia-related AEs occurred in 40.0 % of patients with acromegaly treated with pasireotide SC, and 10.0 % discontinued treatment because of hyperglycemia. Ongoing studies evaluating pasireotide LAR in patients with CD and management of pasireotide-induced hyperglycemia in patients with CD or acromegaly (ClinicalTrials.gov identifiers NCT01374906 and NCT02060383, respectively) will address these key safety issues.

**Conclusions:**

Disease pathophysiology, drug formulation, and physician experience potentially influence the differences in reported rates of pasireotide-induced hyperglycemia in CD and acromegaly. Hyperglycemic effects associated with pasireotide have a predictable pattern, can be managed with antidiabetic agents, and are reversible upon discontinuation.

## Introduction

Cushing’s disease (CD) and acromegaly are rare diseases characterized by the increased production of adrenocorticotropic hormone (ACTH) in the former and growth hormone (GH) in the latter [1]. In CD, hypercortisolemia is caused by the secretion of ACTH from a corticotroph pituitary tumor. In acromegaly, GH excess, most commonly from a somatotroph pituitary tumor, causes downstream hypersecretion of insulin-like growth factor 1 (IGF-1). Chronic elevation of cortisol (CD) and GH and IGF-1 (acromegaly) levels is associated with increased mortality risk and worsening comorbidities in both conditions [1, 2]. Thus, in both diseases, the treatment goals include normalization of hormone levels, reversal of clinical features, prevention of disease recurrence, and reduction in tumor volume in cases of pituitary macroadenomas [1, 3].

Surgery is considered first-line treatment and provides adequate control of cortisol levels in many patients with CD (65–90 %) [4] and GH and IGF-1 levels in patients with acromegaly (microadenomas, >85 %; macroadenomas, 40–50 %) [3]. Despite the success rates associated with surgery, 5–10 % of patients with CD [4] and 2–8 % of patients with acromegaly [3] will experience disease recurrence within 5 years after achieving postoperative hormone remission. Medical therapy (sometimes used as a bridge until adjuvant radiation therapy has taken full effect) is an option for patients with recurrent or persistent CD or acromegaly following surgery.

Pasireotide is a new-generation, multireceptor-targeted somatostatin receptor ligand (SRL) approved for the treatment of patients with CD (subcutaneous [SC] formulation) [5] or acromegaly (long-acting release [LAR] formulation) [6]. It is indicated for patients who do not achieve control with surgery and/or for whom surgery is not an option [5, 6]. Pasireotide binds to several known somatostatin receptor (sst) subtypes that are expressed by corticotroph and somatotroph tumors, resulting in decreased secretion of ACTH and GH, respectively. Both corticotroph and somatotroph tumors express relatively high levels of sst_2_ and sst_5_ [7]. Compared with octreotide, pasireotide binds with higher affinity to sst_1_ (30-fold), sst_3_ (5-fold), and sst_5_ (39-fold) and lower affinity to sst_2_ (3-fold) [8, 9]. Pasireotide also binds with higher affinity than lanreotide to sst_1_ (19-fold), sst_3_ (9-fold), and sst_5_ (106-fold) but with lower affinity to sst_2_ (2-fold) [8]. The combination of the broad binding profile of pasireotide with its preferential binding to sst_5_ provides therapeutic rationale for its use in the treatment of CD and acromegaly. Although its efficacy in clinical studies has warranted consideration of pasireotide as the first treatment of choice among SRLs, the effect of pasireotide on glucose metabolism should be considered when choosing the best SRL option for each patient [10]. This review provides a summary of data from clinical studies and case reports that document hyperglycemia-related adverse events (AEs) associated with pasireotide in patients with either acromegaly or CD.

## Clinical efficacy of pasireotide in CD and acromegaly

### Pasireotide SC in CD

In a phase 3 study of 162 patients with persistent or recurrent CD or newly diagnosed disease, who were not eligible for surgery, twice-daily injections of pasireotide SC provided rapid reductions in mean levels of urinary-free cortisol (UFC) that were sustained during treatment [11]. After 12 months of pasireotide 600 and 900 µg twice daily, 13 and 25 % of patients achieved UFC levels at or below the upper limit of the normal range, respectively. At 12 months, tumor volume reductions were also observed with 600 µg (−9.1 %) and 900 µg (−43.8 %). Decreases in mean UFC were associated with improved signs and symptoms of disease, including systolic blood pressure (mean change from baseline, −6.1 mm Hg), diastolic blood pressure (mean change from baseline, −3.7 mm Hg), weight (mean change from baseline, −6.7 kg), and health-related quality-of-life score (mean change from baseline, 11.1 points). Improvements in facial rubor and supraclavicular and dorsal fat pads were also reported. In addition, 69 % of patients (n = 11) who remained in the study for 60 months achieved UFC levels at or below the upper limit of the normal range [12].

### Pasireotide in acromegaly

In a phase 3 study of patients with acromegaly who were naive to medical therapy, significantly more patients achieved biochemical control (GH < 2.5 ng/mL and age- and sex-adjusted IGF-1) after 1 year of monthly injections of pasireotide LAR 40 mg than after 1 year of monthly injections of octreotide LAR 20 mg (31.3 vs 19.2 %) [13]. Tumor volume reductions were similar between the 2 treatment groups (pasireotide LAR, 40 %; octreotide LAR, 38 %), and both therapies improved symptoms of disease (e.g., perspiration, fatigue, osteoarthralgia, paresthesia, headache) and quality of life.

In another phase 3 study that enrolled patients with acromegaly who were inadequately controlled by other SRLs, monthly injections of pasireotide LAR 40 mg (15 %) and 60 mg (20 %) provided greater biochemical control than the active control treatment (octreotide LAR 30 mg or lanreotide Autogel 120 mg; 0 %) at 6 months [14]. Furthermore, more patients in the pasireotide LAR 40 and 60-mg groups achieved tumor volume reductions of >25 % than did those in the active control group (18.5 and 10.8% vs 1.5 %). Improvements in symptom severity scores also occurred at higher degrees in patients in the pasireotide LAR 40 and 60-mg groups than in those in the active control group. Symptoms included headache, fatigue, perspiration, paresthesia, and osteoarthralgia.

The efficacy and safety of pasireotide SC in acromegaly have also been investigated. In a phase 2 extension study, 33 % of patients (3 of 9) achieved biochemical control after 24 months of treatment [15]. Up to 75 % of patients who responded to treatment demonstrated tumor volume reductions of ≥20 %. Additionally, the number of patients showing improvements in symptoms of disease (i.e., headache, fatigue, perspiration, osteoarthralgia) approximately doubled during the course of the study.

## Hyperglycemia associated with pasireotide in CD and acromegaly

### Clinical trials experience

In clinical trials, the safety profile of pasireotide included AEs, mostly related to gastrointestinal symptoms and cholelithiasis, that were largely consistent with other SRLs [5, 6]. However, compared with other SRLs, treatment with pasireotide is associated with a higher frequency of hyperglycemia in patients with CD or acromegaly [11, 13, 14]. The reported rates of hyperglycemia-related AEs and the percentage of patients who discontinued therapy from such events differ across clinical studies of pasireotide in CD and acromegaly (Table 1). Further comparison of the rates shows that the frequency of hyperglycemia-related AEs was lower in patients with acromegaly who received pasireotide LAR (57.3–67.0 %) [13, 14] than in patients with CD who received pasireotide SC (68.4–73.0 %) [11, 16]. Interestingly, fewer patients with acromegaly who received pasireotide LAR discontinued treatment because of hyperglycemia-related AEs, as reported in 2 separate trials (3.4 and 4.0 %), [13, 14] compared with patients with CD who received pasireotide SC, also as reported in 2 separate trials (6.0 and 5.3 %) [11, 16]. In a clinical study that evaluated the efficacy and safety of pasireotide SC in acromegaly, hyperglycemia-related AEs occurred in 40.0 % of patients, and 10.0 % of patients discontinued treatment because of such events [15].

**Table 1:** Hyperglycemia-related AEs associated with pasireotide in clinical studies of Cushing’s disease or acromegaly

Study	Disease	Study description	Study duration	Drug formulation (dosing)	Hyperglycemia-related AEs	Discontinuations due to hyperglycemia-related AEs (%)
Colao et al. [11]	Cushing’s disease	Phase 3	12 months	Pasireotide SC (600, 900 µg b.i.d)	73.0 %	6.0
Boscaro et al. [16]	Cushing’s disease	Phase 2, extension	6 months	Pasireotide SC (600 µg b.i.d; 900 µg with suboptimal control)	68.4 %	5.3
Colao et al. [13]	Acromegaly	Phase 3	12 months	Pasireotide LAR (40 mg; dose increase to 60 mg with suboptimal control)	57.3 %	3.4
Gadelha et al. [14]	Acromegaly	Phase 3	6 months or longer	Pasireotide LAR (40, 60 mg)	40 mg, 67.0 % 60 mg, 61.0 %	4.0
Petersenn et al. [15]	Acromegaly	Phase 2, extension	6 months or longer	Pasireotide SC (200, 400, 600 µg; 900 µg with suboptimal control)	40.0 %	10.0

*AE* adverse event, *b.i.d* twice daily, *LAR* long-acting release, *SC* subcutaneous

**Fig. 1:**
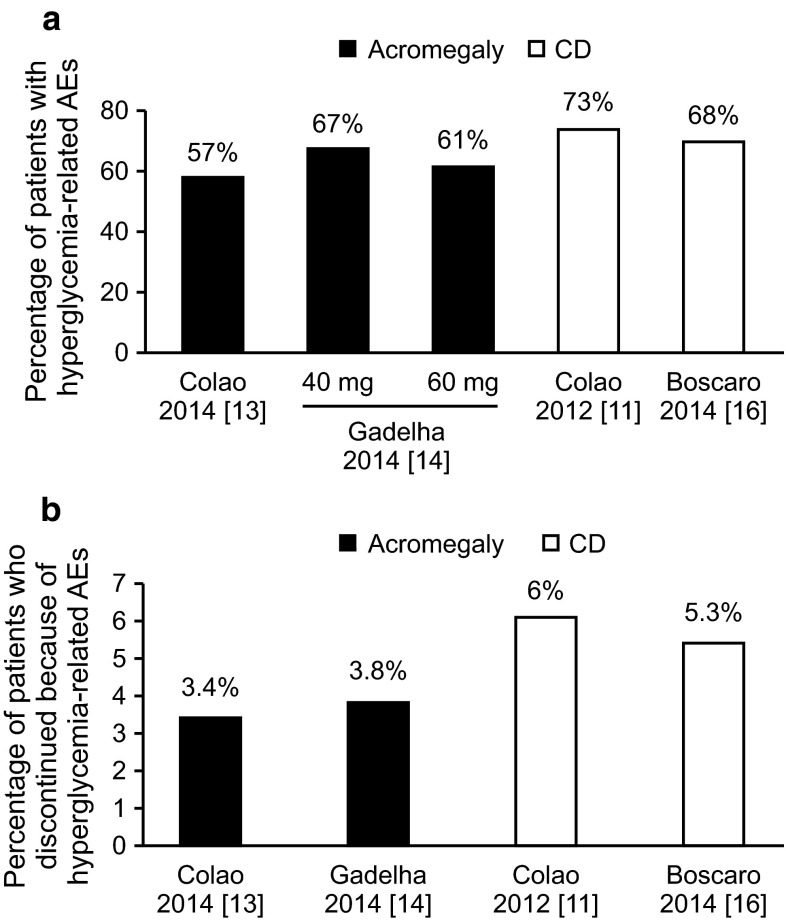
Factors potentially affecting reported differences in **a** the frequency of pasireotide-induced hyperglycemia and **b** the frequency of discontinuations associated with hyperglycemia among patients with CD or acromegaly. *AE* adverse event, *CD* Cushing’s disease, *LAR* long-acting release, *SC* subcutaneous. Figure was created with Adobe Illustrator CC 2015

Several underlying factors could contribute to differences in the reported frequency of pasireotide-induced hyperglycemia among patients with CD or acromegaly. In clinical studies of acromegaly, the rates of pasireotide-induced hyperglycemia-related AEs were lower than those in CD (Fig. 1a). This could be related to disease pathophysiology since the dysregulation of glucose metabolism in CD and acromegaly could be uniquely linked to downstream effects associated with chronic exposure to elevated levels of cortisol or GH/IGF-1, respectively [17, 18]. Also, because studies of pasireotide in CD occurred before those in acromegaly, the benefit of physician experience could have led to lower rates of study discontinuation in the acromegaly trials (Fig. 1b). This could be attributed to a better understanding of the mechanisms underlying pasireotide-induced hyperglycemia and its optimal management in patients with acromegaly or CD. Despite the higher frequency of hyperglycemia-related AEs associated with pasireotide than with other medical therapies for acromegaly or CD, results from other clinical studies suggest that the hyperglycemic effect associated with treatment has a predictable pattern, can be managed with antidiabetic medications (ADM), and is reversible upon discontinuation of treatment with pasireotide.

### Mechanisms of pasireotide-induced hyperglycemia

It has been reported that impaired glucose metabolism observed in patients with CD or acromegaly is uniquely associated with disease pathophysiology. Chronic exposure to elevated GH and IGF-1 levels is associated with insulin resistance, which may be counteracted by the compensatory hyperfunction of pancreatic beta cells in patients with acromegaly with normal glucose tolerance [18]. Similarly, in CD, chronic hypercortisolism blocks the binding of insulin to peripheral tissues, resulting in insulin resistance, and inhibits the release of insulin by pancreatic beta cells [17]. Excess cortisol can also affect glucose metabolism in hepatic tissue by stimulating gluconeogenesis or indirectly inhibiting insulin sensitivity by depleting storage of hepatic glycogen.

Recently, there has been a greater understanding of the mechanisms that underlie the development of hyperglycemia in patients with acromegaly or CD treated with pasireotide. The hyperglycemic effects of pasireotide are primarily due to its tendency to reduce insulin and incretin secretion [19]. Insulin secretion is mediated in large part by sst_2_ and sst_5_ [20, 21], and glucagon secretion is mediated primarily by sst_2_ [21, 22]. Pasireotide binds to sst_2_ and sst_5_ and binds with highest affinity to sst_5_, which is expressed not only by pituitary cells but also by other cell types. For example, pasireotide binds to sst_5_ in pancreatic islet cells, which leads to reduced insulin secretion that is not observed with SRLs that bind to sst_2_ with greater affinity [19, 23]. Reduced insulin levels associated with pasireotide are unable to counterbalance the reduced insulin sensitivity caused by uncontrolled acromegaly or CD [18]. However, two mechanisms could explain why the hyperglycemic effect is transient. First, pasireotide has minimal effects on glucagon secretion and no effects on insulin sensitivity in healthy volunteers [19]. In contrast, octreotide and lanreotide suppress glucagon secretion, which could be due to increased binding of sst_2_ [21, 22], a key mediator of glucagon secretion to which octreotide and lanreotide bind with greater affinities than pasireotide [8]. By improving biochemical control, it is likely that insulin sensitivity will increase, which should improve glucose tolerance, even if insulin secretion remains reduced. Second, it was reported that levels of IGF-binding protein 2 increased after 24 weeks of pasireotide LAR [24], which could attenuate the long-term hyperglycemic effects of pasireotide LAR.

### Levels of fasting plasma glucose and glycated hemoglobin during treatment course

Fasting plasma glucose (FPG) and glycated hemoglobin (HbA_1c_) levels are parameters used to assess glycemic control [25]. In a clinical study of patients with acromegaly, it was reported that up to 45 % of patients with baseline FPG between 100 and 126 mg/dL had FPG levels ≥126 mg/dL after 26 months of pasireotide LAR [26]. In a clinical study of patients with CD treated with pasireotide, worsened diabetic status was observed in 76 % of patients who had normal HbA_1c_ levels at baseline [27]. During the course of treatment in patients with acromegaly or CD, mean FPG levels peaked after 1 month of pasireotide and thereafter remained stable or slightly decreased [11, 12, 14, 26–28]. In some patients, levels of FPG and HbA_1c_ were stabilized during the course of treatment with pasireotide LAR for up to 26 months [26, 27]. These observations suggest that pasireotide initially induces an increase in FPG and HbA_1c_ levels, which then stabilize during the course of treatment.

### Predictive risk factors of pasireotide-induced hyperglycemia

In CD, the prevalence of impaired glucose tolerance or diabetes mellitus at diagnosis is 21–64 % and 20–47 %, respectively [29]. Similarly, the prevalence of impaired fasting glucose and diabetes are higher in patients with acromegaly compared with the general population, predisposing patients to a greater risk of developing hyperglycemia [30]. This suggests that baseline glycemic status before the initiation of pasireotide treatment could be predictive of the extent and severity of hyperglycemia associated with treatment. In a phase 3 study of patients with acromegaly, baseline FPG > 100 mg/dL correlated with the development of higher FPG and HbA_1c_ levels and a higher degree of hyperglycemia during pasireotide LAR treatment [24]. Moreover, it was reported that increasing the dose of pasireotide LAR from 40 to 60 mg was associated with a 21–36 % increased risk of developing hyperglycemia [31, 32]. Regarding disease control as a predictive factor, reductions in GH and IGF-1 in patients with newly diagnosed acromegaly who were treated with octreotide for 6 months without ADM were shown to correlate with change in HbA_1c_ levels [33]; however, a similar association in patients treated with pasireotide has not yet been studied. Other predictive risk factors associated with developing hyperglycemia in patients with acromegaly treated with pasireotide LAR include body mass index ≥25 kg/m^2^, diabetes at baseline, and history of dyslipidemia [34]. Because certain populations are at greater risk of hyperglycemia associated with pasireotide, proactive management and monitoring of these patients are critical for improving clinical outcomes.

## Management of pasireotide-induced hyperglycemia

### Optimal treatment with ADMs

As previously discussed, in phase 3 studies of pasireotide SC in CD and pasireotide LAR in acromegaly, the majority of hyperglycemia-related AEs were mild to moderate in severity [11, 13, 14]. Even in patients with acromegaly with FPG > 250 mg/dL at baseline, ADM initiated within 2 weeks of the first dose of pasireotide LAR led to rapid decreases in FPG, suggesting that early treatment intervention with ADMs could optimally manage pasireotide-induced hyperglycemia [34]. Metformin has been shown to be minimally effective in reducing pasireotide-induced hyperglycemia in healthy volunteers [35]; however, this study measured effects on glucose metabolism after only 6 days, which may have been too brief to observe long-term effects of metformin. For instance, metformin has been shown to increase glucagon-like peptide 1 (GLP-1) [36], which may at least partially compensate for the reduction in GLP-1 associated with pasireotide [19]. Antidiabetic medication, primarily metformin, was administered in 38–48 % of patients in acromegaly trials [13, 14, 26]. Mean HbA_1c_ levels in patients with acromegaly naive to medical therapy who developed pasireotide-induced hyperglycemia were <7 % after 12 months when treated with metformin alone or in combination with other ADMs [37]. A case study of a patient with CD exemplified that control of hyperglycemia could be achieved with metformin in combination with glipizide and sitagliptin [38]. These results suggest that metformin is useful for some patients in treating pasireotide-associated hyperglycemia.

In some case study reports, treatment with metformin alone was insufficient in providing glycemic control to patients with pasireotide-induced hyperglycemia [39–41]. In these cases, treatment with insulin alone or glipizide with or without insulin was able to quickly reduce FPG levels. Dietary control or modification could also be important for management of pasireotide-induced hyperglycemia [16, 42]. In healthy volunteers who were administered pasireotide SC, coadministration with liraglutide, a GLP-1 agonist, was most effective in reducing serum glucose levels (72 % lower than with pasireotide alone) [35]. Coadministration with vildagliptin, a dipeptidyl peptidase-4 (DPP-4) inhibitor, was most effective in attenuating the reduction in serum insulin levels (71 % higher than with pasireotide alone). These studies indicate that by following standard guidelines for the treatment of diabetes and adapting treatment with ADMs on the basis of each patient’s glycemic status, pasireotide-induced hyperglycemia can be effectively managed.

### Treatment recommendations

Medical expert recommendations have been established for the management of pasireotide-induced hyperglycemia in patients with CD [17, 43]. In patients not currently treated with insulin, and particularly for those with insulin resistance, it is recommended that metformin be initiated or continued if FPG > 126 g/dL or HbA_1c_ is >6.5 % [43]. In cases in which patients fail to achieve glycemic control with metformin, it is suggested that a DPP-4 inhibitor be added. A GLP-1 receptor agonist should replace the DPP-4 inhibitor if HbA_1c_ remains >7.0 %. If neither DPP-4 inhibitors nor GLP-1 receptor agonists provide glucose control, it is suggested that treatment with insulin be initiated while maintaining metformin treatment. Although no specific recommendations regarding pasireotide-induced hyperglycemia in patients with acromegaly have been published, treatment with metformin plus a DPP-4 inhibitor, GLP-1 agonist, or insulin would most likely have similar efficacy.

### Monitoring recommendations

Patients with CD or acromegaly who initiate, discontinue, or need dose adjustment of pasireotide should be proactively managed in accordance with current monitoring guidelines and recommendations. Levels of FPG and HbA_1c_ should be evaluated before initiation of treatment, and blood glucose should be monitored weekly for the first 2–3 months after initiation and for the first 2–6 weeks after a dose increase [5, 6]. Similarly, medical expert opinion also recommends that, in patients with normal glucose metabolism prior to initiating pasireotide, fasting and postprandial glucose levels should be monitored several times per day, twice during the first week, and once weekly thereafter [17, 43]. Moreover, patients with abnormal glucose metabolism during treatment should be monitored daily upon initiating pasireotide and while adjusting treatment as necessary to optimally control glycemic levels [5, 6, 17, 43]. If warranted, dose reduction or discontinuation of pasireotide should be implemented if hyperglycemia is not controllable despite medical management [5, 6]. It is suggested that FPG and HbA_1c_ levels of patients who discontinue pasireotide may need to be assessed frequently to reduce the risk of developing hypoglycemia.

## Reversibility of hyperglycemia upon discontinuation of pasireotide

Clinical studies of pasireotide in healthy volunteers and in patients with CD or acromegaly suggest that the hyperglycemic effects associated with pasireotide have a predictable pattern and could be effectively managed by following recommended guidelines set forth by expert opinions [5, 6, 17, 43]. Moreover, deterioration in glycemic status associated with pasireotide is reversible upon treatment discontinuation, which potentially restores glycemic parameters to normoglycemic levels [44]. The reversibility of the hyperglycemic effects of pasireotide has been shown in a pharmacokinetic analysis of single-dose administration, in which mean glucose levels increased to ≥200 µg and then normalized 23 h after injection [45]. In a previous case study, normalization of glycemic levels was demonstrated in a patient who discontinued pasireotide SC because of lack of response to treatment [44]. In a phase 3 clinical trial of patients with acromegaly who were treated with pasireotide, mean FPG and mean HbA_1c_ levels were reduced to 104 mg/dL (mean value at baseline, 127 mg/dL) and to 6.12 % (mean value at baseline, 6.71 %), respectively, within 3 months of switching from pasireotide LAR to octreotide LAR [28]. In conclusion, hyperglycemia associated with pasireotide is transient and not expected to cause long-term effects if pasireotide is discontinued.

## Conclusions

Pasireotide is a viable medical therapy option for patients with CD or acromegaly but is associated with higher rates of hyperglycemia and diabetes mellitus than other medical therapies. Further research is needed to determine optimal treatment strategies with ADMs to achieve glycemic control in patients with pasireotide-induced hyperglycemia. For example, a multivariate analysis could be performed of baseline FPG and HbA_1c_ levels across clinical studies of pasireotide in CD or acromegaly to further assess the predictability and manageability of pasireotide-induced hyperglycemia. A phase 4 study is currently ongoing that will evaluate the effects of incretin-based therapy compared with insulin on glycemic control at 16 weeks in patients with CD or acromegaly (ClinicalTrials.gov identifier NCT02060383) [46]. Significant progress has been made over the years to better understand the mechanisms by which impaired glucose metabolism in patients is uniquely linked to the disease pathophysiology of CD and acromegaly and how pasireotide modulates insulin secretion and sensitivity. There is growing evidence to demonstrate that proactive management with ADMs can effectively manage hyperglycemia-related AEs associated with pasireotide during the course of treating patients with CD or acromegaly.
